# *Pediococcus pentosaceus* Enhances Host Resistance Against Pathogen by Increasing IL-1β Production: Understanding Probiotic Effectiveness and Administration Duration

**DOI:** 10.3389/fimmu.2021.766401

**Published:** 2021-11-26

**Authors:** Chengjie Shan, Miao Li, Zhu Liu, Rong Xu, Fang Qiao, Zhen-Yu Du, Mei-Ling Zhang

**Affiliations:** ^1^ Laboratory of Aquaculture Nutrition and Environmental Health, School of Life Sciences, East China Normal University, Shanghai, China; ^2^ School of Basic Medical Sciences, Shanghai University of Traditional Chinese Medicine, Shanghai, China

**Keywords:** probiotic, pathogen resistance, administration duration, butyrate, NLRP3 inflammasome

## Abstract

Probiotic administration is a potential strategy against enteric pathogen infection in either clinical treatment or animal nutrition industry, but the administration duration of probiotics varied and the underlying mechanisms remain unclear. A strain (YC) affiliated to *Pediococcus pentosaceus*, a commonly used probiotic, was isolated from fish gut and the potential role of YC against *Aeromonas hydrophila* was detected in zebrafish. We found that 3- or 4-week YC administration (YC3W or YC4W) increased the resistance against *A. hydrophila* while 1- or 2-week treatment (YC1W or YC2W) did not. To determine the possible reason, intestinal microbiota analysis and RNAseq were conducted. The results showed that compared with CON and YC1W, YC4W significantly increased the abundance of short-chain fatty acids (SCFAs) producing bacteria and elevated the gene expression of *nlrp3*. Higher butyrate content and enhanced expression of IL1β were subsequently found in YC4W. To identify the causal relationship between butyrate and the higher pathogen resistance, different concentrations of sodium butyrate (SB) were supplemented. The results suggested that 10 mmol/kg SB addition mirrored the protective effect of YC4W by increasing the production of IL-1β. Furthermore, the increased IL-1β raised the percentage of intestinal neutrophils, which endued the zebrafish with *A. hydrophila* resistance. *In vivo* knockdown of intestinal *il1b* eliminated the anti-infection effect. Collectively, our data suggested that the molecular mechanism of probiotics determined the administration duration, which is vital for the efficiency of probiotics. Promoting host inflammation by probiotic pretreatment is one potential way for probiotics to provide their protective effects against pathogens.

## Introduction

Probiotic supplementation is commonly used to protect the host against enteropathogenic bacteria in human or animals and the mechanisms of different probiotics varied (1). Probiotics increased the host resistance against pathogen infection and could be attributed to host immunomodulation-independent and immunomodulation-dependent types (2). In the immunomodulation-independent type, probiotics mainly inhibit pathogens by nutrient competition, niche occupancy, or bacteriocin production (3–5). In the immunomodulation-dependent type, probiotics could modulate the immune responses of the host in direct (bacterial compounds or metabolites) or indirect (gut microbiota modulation) ways (6). It has been reported that some probiotic strains including *Lactobacillus* and *Bacillus* directly inhibit pathogen adhesion by producing bacteriocin or interfering quorum sensing (7, 8), while others could modulate the gut microbiota shift to a healthier status to protect the host. For example, *Lactobacilli* has been reported to protect the pig from pathogen invasion by improving the abundance and number of *Lactobacilli* and other indigenous probiotic bacteria (9). *Bacillus clausii* is reported to control the development of *Clostridium difficile* infection in clinical trials and the anti-infection activity of *B. clausii* is gut microbiota dependent (10). *Faecalibacterium prausnitzii* ameliorated gut dysbiosis by increasing the abundance of short-chain fatty acids (SCFA)-producing bacteria (11). All these researches suggested that regulating the intestinal microbiota is an effective way of probiotics to protect the host.

*Pediococcus pentosaceus* is one common probiotic and multiple strains have been reported to play antibacterial roles in hosts (12, 13). *P. pentosaceus* has been applied in food industry (14) and used as a growth promoter in animal nutrition (15). *P. pentosaceus* can produce bacteriocins to directly inhibit pathogen proliferation, and it can also regulate intestinal bacteria to protect the host against pathogens (16, 17). For example, oral administration of *P. pentosaceus* LI05 for 14 days enhanced the survival rate of *C. difficile* infected mice by increasing the abundance of beneficial microbial taxa and restraining the opportunistic pathogens in mice gut (18). In a recent study, 30-day pretreatment of *P. pentosaceus* SL001 is reported to alter the intestinal microbiota composition and upregulate the expression level of immune-related genes to protect the carp against *A. hydrophila* infection (19). *P. pentosaceus* was also reported to elevate the intestinal propionate and butyrate by increasing the relative abundance of SCFA-producing bacteria (20). However, the exact mechanism by which *P. pentosaceus* play its protective effect against pathogens remains unclear.

Pattern recognition receptors (PRR) are important in maintaining the intestinal homeostasis (21). NOD-like receptors (NLRs) are known for pathogen-associated molecular pattern (PAMP) recognition and play essential roles in the innate immune response against pathogen infection. When sensing PAMPs, NLRs were activated to increase the expression of pro-inflammatory cytokines. Among all NLRs, the activation of the pyrin domain-containing NLRP3 recruits adaptor protein ASC and caspase-1 for NLRP3 inflammasome formation, which results in IL-1β maturation and secretion (22). Higher expression of IL-1β could enhance the migration and recruitment of neutrophils in the inflammation or infected sites (23). Previous studies suggested that probiotics suppressed the activation of the inflammasome and reduced the inflammation caused by pathogen infection (24), while probiotic is also reported to trigger the activation of the inflammasome; for example, incubation of Caco-2 cells with probiotic *Escherichia coli* Nissle 1917 resulted in lower inflammasome activation and subsequent secretion of IL-18 (25). An *in vivo* study showed that *Lactobacillus reuteri* B1/1-treated chicken exhibited higher gene expression of NLRP3 and higher *Campylobacter jejuni* resistance (26), but how the activated inflammasome increased pathogen resistance still needs more evidence.

The duration of treatment is important for probiotics to exert their beneficial effects. It was reported that 6–8 h *Enterococcus faecium* NCIMB 10415 pretreatment significantly increased the transepithelial electrical resistance of Caco-2 cells *in vitro*, while a shorter (<6 h) or longer (>8 h) treatment did not (27). It was found that compared with the control group and 1-week treatment group, 2-week *Kocuria* SM1 addition significantly reduced the mortality of rainbow trout challenged with *Vibrio anguillarum* (28). Different administration duration influences the probiotics efficiency and the underlying mechanism should be addressed.

Zebrafish is an excellent model for studying host–gut microbe–pathogen interactions because it is optical transparency during early development status and it shares the most orthologous genes with mice (29, 30). Furthermore, it can be used for generating a series of mutant lines in a short time because its rapid reproduction and it has a conserved innate immune system with mammals (31). Nowadays, functions of many probotics, including *Lactobacillus* sp., *Bacillus* sp., and *Bifidobacterium* sp., have been investivated in zebrafish (32).

In the present study, a bacterial strain (YC) affiliated to *P. pentosaceus* was used to administrate zebrafish for up to 4 weeks. *Aeromonas hydrophila*, a commonly used pathogen for fish, was used to infect zebrafish. The pathogen-resistant effect of YC was detected and the possible mechanism was identified. We found that 4-week but not 1-week YC administration increased the level of intestinal butyrate and subsequently increased the production of IL-1β *via* NLRP3 inflammasome activation, which contributed to the higher resistance against *A. hydrophila* of zebrafish.

## Materials and Methods

### Ethics Statement

All experiments were carried out according to the guidelines for the care and use of laboratory animals in China. This work was approved by the Committee on the Ethics of Animal Experiments of East China Normal University (assurance no. F20201002).

### Bacteria and Culture Condition

A strain (designated as YC) was isolated from the intestine of Nile tilapia by using MRS broth (De Man, Rogosa, Sharpe) at 28°C. Briefly, the gut contents of Nile tilapia were collected and diluted with PBS. After 700×*g* centrifugation, the supernatant was inoculated on the MRS agar plate. According to the 16S rRNA sequencing, the most enriched bacteria were affiliated to *P. pentosaceus*. One clone was randomly picked and designated as YC. The phylogenetic analysis was performed with MEGA 7.0. The nearest neighbors of YC were identified by 16S rRNA gene sequencing (BioProject PRJNA732367) and RDP SEQMATCH. *A. hydrophila* ATCC 7966 was purchased from China General Microbiological Culture Collection Center (resource no. 1511C0002100007469), and cultured in LB broth at 37°C, 180 rpm. Bacteria were centrifuged at 3,000 rpm for 10 min at 4°C and resuspended in PBS to a final concentration of 10^9^ CFU/ml before the administration.

### Zebrafish Husbandry and Experimental Diet

Adult zebrafish (3 months old) line AB (male:female = 1:1) were obtained from the Chinese National Zebrafish Resource Center (Wuhan, China). Zebrafish maintenance and breeding were conducted according to the standard methods (33). The water pH was maintained at 7.0 to 8.0 and the temperature was maintained at 28°C. The dissolved oxygen in the water was 5–8 mg/L. In the YC administration trial, zebrafish were randomly divided into five experimental groups and each group has three replicate tanks in a partial-reuse system. YC was administrated in water at a concentration of 10^6^ CFU/ml for 0 week (CON), 1 week (YC1W), 2 weeks (YC2W), 3 weeks (YC3W), and 4 weeks (YC4W). The water was renewed (50%) every day. YC was re-provided after water renewal. The fish were maintained under a 14-h day and 10-h dark photoperiod.

For the sodium butyrate supplementation trial, zebrafish were fed with a commercial diet containing 50% protein and 8% lipid (Shengsuo, China) supplemented with 10 (SB10), 20 (SB20), and 40 mmol/kg (SB40) sodium butyrate (Sigma, USA) for 7 days using a spraying method. Zebrafish fed with a commercial diet were designed as the control group. Zebrafish were fed with the diets at a ratio of 4% of their average body weight twice daily at 10:00 a.m. and 18:00 p.m. during the feeding period.

### Sample Collection

After each feeding trail, five adult zebrafish (3 months old) from each treatment were randomly sampled and dissected under MS222 (325 mg/L) anesthesia. The whole intestinal contents were collected for short-chain fatty acids analysis and 16S rRNA gene sequencing. The whole intestine was collected for the gene expression level detection, Western blot, RNAseq, and flow cytometry analysis. All samples were kept at −80°C until use.

### *P. pentosaceus* YC Labeling With FDAA Probes and Transplantation

*P. pentosaceus* YC was cultured in MRS medium at 28°C till the mid-exponential phase before labeling. Then, TAMRA (tetramethylrhodamine)-bearing FDAA (TADA) was added to the medium to a final concentration of 300 µM (34). The bacteria were labeled for 6 h and added to the water at a final concentration of 10^6^ CFU/ml. Five zebrafish larvae at 4 days post-fertilization (dpf) were then immersed in the water for 24 h. Zebrafish larvae were anesthetized using MS-222 (Sigma, USA) at a concentration of 325 mg/L and washed three times before the fluorescence microscopy analysis. Confocal images were obtained using a Multiphoton Microscope Leica TCS SP8 DIVE (Leica, Germany) with a 555-nm laser. Images were processed by Imaris (Bitplane, UK) and ImageJ (NIH, USA) software.

### Challenge Test

At the end of the experiment, adult zebrafish (3 months old, 30 fish/group, three replicates per tank) were bathed in the water (28°C, pH 7.0–8.0) containing 10^8^ CFU/ml *A. hydrophila* according to the method previously described (35), and the mortality was recorded for 7 days. For the *in vivo il1b* siRNA trial, zebrafish were challenged with *A. hydrophila* at a concentration of 10^9^ CFU/ml, and the mortality was recorded for 48 h. Each curve represents the sum of three independent tanks.

### Gut Microbiota Analysis

At the end of the YC administration experiments, 30 fish from each group underwent anesthetization using MS-222 (Sigma, USA) at a concentration of 325 mg/L. The intestinal contents of five zebrafish were pooled as one sample and total bacterial genomic DNA was isolated from the mixture of intestinal contents of zebrafish using the OMEGA DNA Kit (Omega, USA). PCR amplification of the bacterial 16S rRNA genes of the V3–V4 region was carried out using the forward primer 338F (5’-ACTCCTACGGGAGGCAGCA-3’) and the reverse primer 806R (5’-GGACTACHVGGGTWTCTAAT-3’). The amplifications were pooled in equal amounts and subsequently submitted for sequencing using an Illumina Miseq platform. Sequencing reads were analyzed with QIIME2 (36). In brief, raw sequence data were demultiplexed using the demux, then the sequences were quality filtered, denoised, merged, and chimera-removed using the DADA2 plugin. Non-singleton amplicon sequence variants (ASVs) were aligned with MAFFT software. Alpha diversity metrics (Chao 1, Shannon, and Simpson) and beta diversity metrics (weighted UniFrac distances) were calculated using the diversity plugin after sequencing depth was downsized to 2,996 sequences per sample. The distance matrices were demonstrated by two-dimensional principal coordinates analysis (PCoA) plots. The raw data were available in GenBank as Sequence Read Archive (SRA) (BioProject PRJNA674514).

### Short-Chain Fatty Acids Quantification

For SCFA analysis, adult zebrafish intestinal content was collected. The intestine samples from five fish were pooled and resuspended with 200 µl of distilled water. Samples were then acidified with 50 µl of 50% sulfuric acid and well mixed using a vortex mixer (DLAB Scientific, China). SCFAs were extracted with 250 µl of diethyl ether and the concentration was measured by a Gas chromatography Nexis GC-2030 (SHIMADZU, Japan). The oven temperature was programmed from 60°C to 100°C at a rate of 5°C/min, with a 2-min hold; to 180°C at 5°C/min, with a 2-min hold. The temperature of the injection port and fame-ionization detector was 200°C. The external standard method was used for SCFA concentration calculation.

### Quantitative PCR

Total RNA was isolated from the whole intestine of zebrafish using the RNAiso Plus (TaKaRa, Japan). Complementary DNA (cDNA) was synthesized using PrimerScript TM RT reagent Kit (TaKaRa, Japan). Quantitative PCR (qPCR) was performed on a QuantStudio^®^ 5 Real-Time PCR Instrument (Applied Biosystems, USA) thermocycler with the following cycle parameters: 94°C for 3 min followed by 40 cycles of 94°C for 10 s and 60°C for 30 s. Gene expression levels were normalized to the expression of elongation factor 1 alpha gene *Ef1a* and β*-actin*. The primer sequences used in the present study are shown in **Table S3**. The relative mRNA expression levels of genes were estimated by using the method of 2^−ΔΔCt^ thereof, ΔCt = Ct_target_ − (Ct_EF1α_ + Ct_β-actin_)/2.

### RNAseq Libraries and RNAseq Analysis

Total RNA was isolated from the whole intestine of zebrafish and mRNA was purified from total RNA using poly-T oligo-attached magnetic beads and divalent cations under elevated temperature for fragmentation. Then, the first- and the second-strand cDNA was synthesized. The library fragments were purified using the AMPure XP system (Beckman, USA) and amplified using Illumina PCR Primer Cocktail in a 15-cycle PCR reaction. Products were quantified by the Agilent High Sensitivity DNA Kit (Agilent, USA) on a Bioanalyzer 2100 system (Agilent, USA). Paired-end sequencing was subsequently performed on a Hiseq platform (Illumina, USA). The raw data were available under the BioProject accession number PRJNA674905.

For RNAseq analysis, the filtered data were obtained by removing adaptor-containing or low-quality reads and then mapped to the *Danio rerio* reference genome (Zv9, Ensembl) using HISAT2 software. HTSeq was used to generate the read count number of each mapped gene. Gene expression level normalization was compiled using Fragments Per Kilobases per Million fragments (FPKM). Differential expression analysis between CON and YC4W, or YC1W and YC4W was conducted using DEGSeq R package (37). *p*-value < 0.05 and |log2 Fold Change| > 1 gene was considered differentially expressed. Differentially expressed genes (DEGs) were functionally assorted and characterized using the Database for Annotation, Visualization and Integrated Discovery (DAVID) v6.8 (38).

### ELISA

The intestinal tissue without intestinal contents of zebrafish from each group was collected and homogenized. The level of IL-1β (JianglaiBio, JL48855-96T) was determined using Zebrafish Interleukin 1β (IL-1 β) Kit according to the manufacturer’s instructions.

### Immunohistochemistry

For zebrafish intestinal immunohistochemistry, OCT (Sakura Finetek, Japan)-embedded frozen sections were fixed using anhydrous methanol (Servicebio, China) for 20 min. Antigen retrieval was carried out using 10 mM sodium citrate (pH 6) in a microwave for 15 min. Endogenous peroxidases were quenched with 3% H_2_O_2_ for 25 min. All slides were blocked with 10% nonspecific antigen with normal rabbit serum (BosterBio, AR1010, USA) for 30 min at room temperature and then incubated with 1:50 diluted cleaved-IL-1β (Affinity Biosciences, AF4006, USA) antibody overnight at 4°C. After that, slides were incubated with 1:200 diluted horseradish peroxidase-conjugated secondary antibodies (Serivcebio, GB23303, China) at room temperature for 30 min and developed with 3,3’-diaminobenzidine (DAB) solution (DAKO, K5007, Denmark). Slides were counterstained with hematoxylin. Images of stained slides were processed by Nikon Eclipse TS100 (Nikon, Japan).

### Western Blotting

The intestine of zebrafish was lysed using RIPA buffer (Beyotime, China) supplemented with protease and phosphatase inhibitor cocktail (Thermo Scientific, USA) for total protein extraction. The concentration was measured using Bicinchoninic acid (BCA) assay Protein Assay Kit (Beyotime, China). Equivalent amounts of total protein were run on an SDS-PAGE gel and then transferred onto a nitrocellulose membrane (Millipore, USA). After 1 h incubation in 5% BSA blocking solution at room temperature, the membranes were incubated with 1:600 diluted anti-GAPDH antibody (Affinity Biosciences, AF0911, USA) or anti-cleaved IL-1 beta antibody (Affinity Biosciences, AF4006, USA) overnight at 4°C. Subsequently, the membranes were washed in TBST and incubated for 1 h at room temperature with 1:10,000 diluted IRDye^®^ 800CW secondary antibody (LI-COR, 925-32211, USA). Visualization was carried out using Odyssey Clx (LI-COR, USA) and the densitometric quantification was performed using Image Studio (LI-COR, USA) software.

### *In Vivo il1b* siRNA in the Zebrafish Intestine

Chemically modified siRNA (**Table S4**) were delivered into the zebrafish intestine using an oral intubation method reported before (39). In brief, zebrafish were anesthetized and placed on a wet sponge. Ten microliters of PBS, non-targeting siRNA, 5 µM, 25 µM, and 50 µM *il1b* siRNA1 (Target site1), and 5 µM, 25 µM, and 50 µM *il1b* siRNA2 (Target site2) were delivered into the zebrafish intestine using a microinjector *via* oral gavage. After 12 and 24 h, the gut was collected for *il1b* quantification.

### Flow Cytometry

For flow cytometry analysis, adult zebrafish (3 months old) intestine was collected for the generation of single-cell suspension according to the protocols reported previously (40, 41). Intestinal single-cell suspension was double-stained with 1:400 diluted FITC anti-CD11b (Biolegend, 101206, USA) and APC anti-Ly6G (Biolegend, 127613, USA) antibodies on ice for 30 min. After washing for three times, cells were resuspended in 2% FBS-containing PBS and analyzed using a CytoFLEX flow cytometer (Beckman coulter, USA).

### Statistical Analysis

All experimental data are presented as the mean ± standard error of the mean (SEM). Data were analyzed for normal distribution using D’Agostino and Pearson normality test and *F* test was used to compare variances. One-way analysis of variance followed by Dunnett’s multiple comparisons test was used to compare the differences among groups and unpaired Student’s *t*-test was used for analysis of differences between groups. Mantel–Cox test was used for analysis of survival curve differences. GraphPad Prism 8 software (GraphPad, USA) was used to perform all statistical analysis.

## Results

### YC Increased the Resistance of Zebrafish Against *A. hydrophila* After a 4-Week Administration

**Figure 1:**
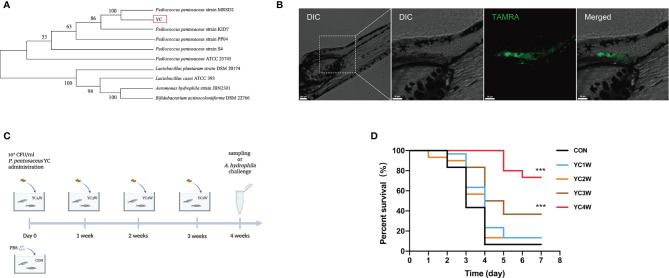
Administration of the isolated bacteria YC and its protective effect on the resistance against *A. hydrophila* of zebrafish. **(A)** Phylogenetic tree of the isolated bacteria bacterium. Distance was calculated by using 16S rRNA gene based on the neighbor-joining criterion and the bootstrap confidence values were 100 replicates. **(B)** Confocal fluorescence imaging of zebrafish larvae (4 dpf) immersed in water containing 10^6^ CFU/ml *P. pentosaceus* YC (pre-treated with 300 µM TAMRA) for 24 h. **(C)** Experimental design for accessing the protective effect of *P. pentosaceus* YC administration. YC was administrated into water at a concentration of 10^6^ CFU/ml for 1 week (YC1W), 2 weeks (YC2W), 3 weeks (YC3W), and 4 weeks (YC4W). PBS treatment as control group (CON). YC or PBS were provided every 24 h for different durations. **(D)** Survival curve of zebrafish from different groups following 10^8^ CFU/ml *A. hydrophila* challenge. Each curve represents the sum of three independent tanks. ***p < 0.001 by Mantel–Cox test.

A bacterial strain (designated as YC) was isolated from the fish intestine and the 16S rRNA gene sequencing showed that the nearest neighbor of the isolated bacterium is *P. pentosaceus* (**Figure 1A**). YC was directly added to the water where the zebrafish were cultured. To determine whether the water-administrated YC could successfully reach the zebrafish gut, YC was labeled with fluorescent D-amino acids (FDAA). The result revealed that YC was successfully detected in the intestine of zebrafish larvae (**Figure 1B**), suggesting that the water-administrated YC could reach the zebrafish intestine.

To determine the protective effect of YC against *A. hydrophila*, zebrafish were treated with YC for different durations from 1 week to 4 weeks (**Figure 1C**). At the end of the trial, the *A. hydrophila* challenge test was conducted. The results indicated that zebrafish of YC4W and YC3W had a significantly higher survival rate compared with CON on the seventh day post *A. hydrophila* challenge while zebrafish from YC2W and YC1W showed no significant difference with CON (**Figure 1D**).

### Influence of YC Administration on the Composition of Gut Microbiota

**Figure 2:**
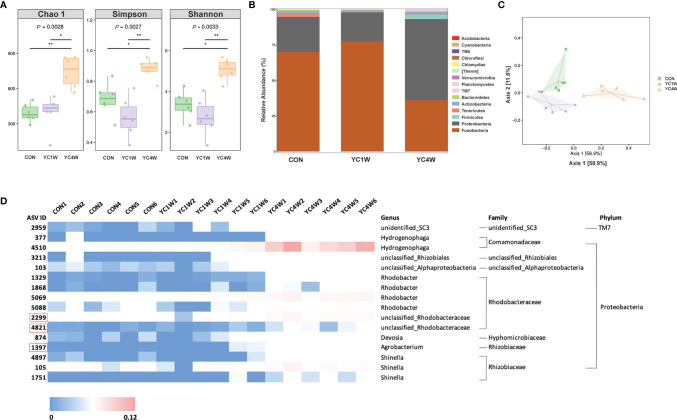
Administration of *P. pentosaceus* YC for 4 weeks alerts gut microbiota composition. 16S rRNA gene sequencing of zebrafish intestinal contents from each group (*n* = 6). **(A)** Alpha diversity metrics, *p*-values were identified with Kruskal–Wallis rank sum test. **(B)** Gut microbiota community of each group at the phylum level. **(C)** Principal coordinate analysis (PCoA) based on Bray–Curtis dissimilarity matrices to show beta diversity among groups at ASV level. **(D)** Heatmap analysis of 16 significantly changed ASVs in the YC4W group compared with the CON and YC1W group. The color bar of each ASV in each treatment is shown. The taxonomy of the ASVs (genus, family, and phylum) is described on the right.

Several studies reported that probiotics exert anti-pathogen benefits by modulating the host–gut microbiota composition (42). Considering the different protective effects between 1-week and 4-week YC administration, samples from CON; YC1W, which has no significant difference with CON in the challenge test; and YC4W, which showed the significant difference with CON in the challenge test, were collected for intestinal bacterial composition analysis. The results showed that 4-week YC administration significantly increased the community richness and diversity of gut microbiota, while YC1W showed no difference compared with CON (**Figure 2A**). At the phylum level, Fusobacteria and Proteobacteria, two dominant phyla in the zebrafish gut, showed no obvious difference between CON and YC1W, while Fusobacteria significantly decreased and Proteobacteria significantly increased in YC4W compared to CON (**Figure 2B**). The ASV-based principal coordinate analysis plot also indicated that samples in YC4W differed from those in CON and YC1W (**Figure 2C**). Compared to CON and YC1W groups, 4-week YC administration significantly increased the proportions of *Hydrogenophaga*, *Rhizobiales*, *Alphaproteobacteria*, *Rhodobacter*, *Rhodobacteraceae*, *Devosia*, *Agrobacterium*, and *Shinella* (**Figure 2D**). In summary, 4-week YC administration alters the zebrafish intestinal microbial community structure.

### Gut Microbiota Alteration Contributes to the Accumulation of Intestinal Butyrate

**Figure 3:**
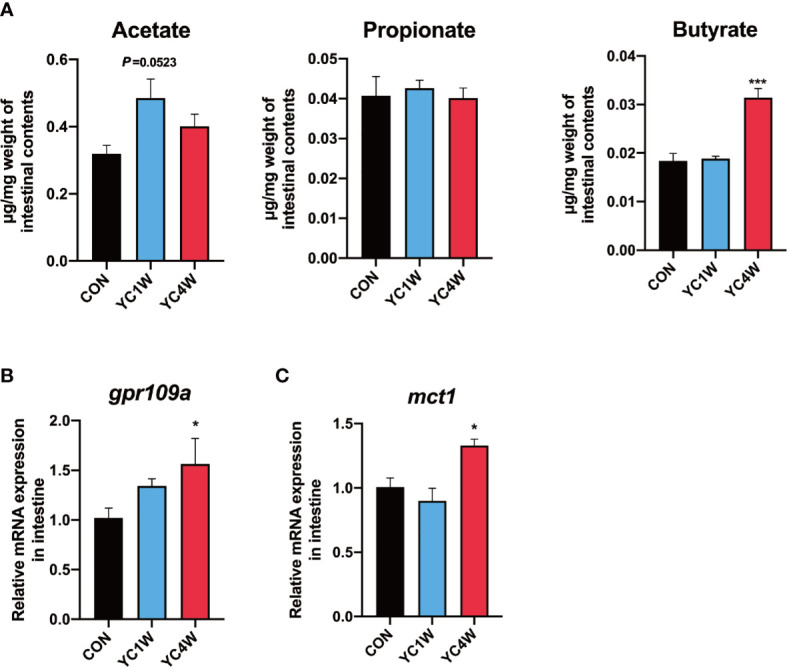
Gut microbiota alteration induces the accumulation of gut butyrate. **(A)** Intestinal short-chain fatty acid levels in CON, YC1W, and YC4W groups (*n* = 3). **(B)** Relative mRNA expression of butyrate-sensing receptor gene *gpr109a* and **(C)** butyrate transporter gene *mct1* in zebrafish intestine (*n* = 6). Data are presented as mean ± SEM. *p < 0.05; ***p < 0.001 by one-way analysis of variance followed by Dunnett’s multiple comparisons test.

Considering *Rhodobacteraceae* and *Agrobacterium*, which were significantly increased in the YC4W group, are closely related to the production of SCFAs (43, 44), SCFA content of zebrafish intestine in CON, YC1W, and YC4W was detected. The results indicated that there was no significant difference in acetate, propionate, and butyrate level between CON and YC1W, while the concentration of butyrate significantly increased in YC4W compared with CON (**Figure 3A**). Consistent with the butyrate content, the expression level of butyrate-sensing receptor *gpr109a* and SCFAs transporter *mct1* was significantly enhanced in YC4W compared with CON but the expression level of these two genes showed no significant difference in YC1W compared with CON (**Figures 3B, C**). We tested the butyrate-producing ability of YC; however, we did not detect an increase in butyrate in YC culture medium (**Figure S1**). Together, our data suggested that 4-week YC administration induced the accumulation of butyrate in the intestine of zebrafish while YC did not produce butyrate *in vitro*.

### The Production of Pro-Inflammatory Cytokine IL-1β Contributes to the Pathogen Resistance of Zebrafish

**Figure 4:**
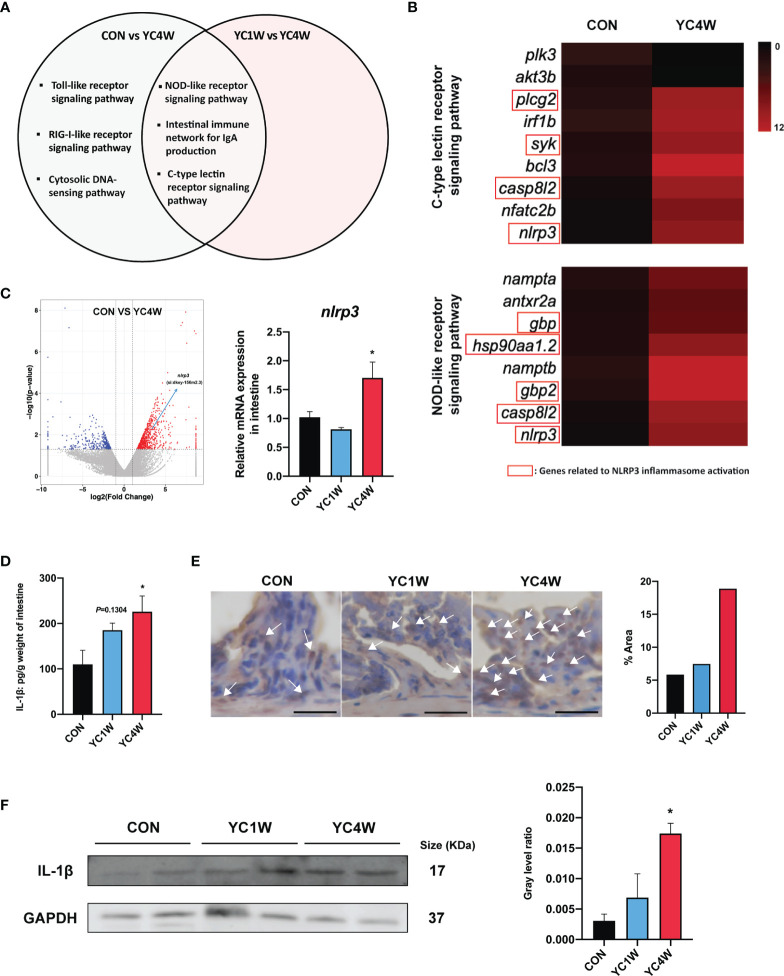
*P. pentosaceus* YC contributes to NLRP3 inflammasome activation after a 4-week administration. Differentially expressed genes (CON *vs*. YC4W, YC1W *vs*. YC4W) of zebrafish intestine were analyzed and used for pathway classification based on KEGG enrichment analysis. **(A)** Immune-related pathways changed in both comparison CON *vs*. YC4W and YC1W *vs*. YC4W. **(B)** Heatmap of differentially expressed genes of C-type lectin receptor signaling pathway and NOD-like receptor signaling pathway in comparison to CON *vs*. YC4W. **(C)** Differentially expressed intestinal genes in comparison to CON *vs*. YC4W and relative *nlrp3* mRNA quantification of zebrafish intestine by qPCR (*n* = 6). **(D)** ELISA analysis of intestinal IL-1β level of zebrafish from CON, YC1W, and YC4W groups (*n* = 6). Representative **(E)** immunohistochemical and **(F)** Western blot analysis of intestinal IL-1β of zebrafish from CON, YC1W, and YC4W groups. Scale bars represents 20 µm. Data are represented as mean ± SEM. *p < 0.05 by one-way analysis of variance followed by Dunnett’s multiple comparisons test.

To investigate the possible mechanism by which YC regulated zebrafish resistance to the pathogen, RNAseq was conducted in CON, YC1W, and YC4W. DEGs in the comparison of CON *vs*. YC4W and YC1W *vs*. YC4W were selected based on KEGG pathway enrichment analysis (**Tables S1, S2**). We screened all immune system-related pathways and found three pathways significantly changed in both comparisons CON *vs*. YC4W and YC1W *vs*. YC4W, including NOD-like receptor signaling pathway, intestinal immune network for IgA production, and C-type lectin receptor signaling pathway (**Figure 4A**). Because the equivalents of IgA gene segments have not been found in zebrafish and IgM is the best-characterized teleost immunoglobulin isotype that represents the prevalent immunoglobulin in systemic immune responses (45), the concentrations of intestinal and serum IgM were measured. However, no significant difference was found among treatments (**Figure S2**). In the other two pathways, we found that the significantly changed genes were involved in NLRP3 inflammasome activation (**Figure 4B**). The expression level of *nlrp3* was measured to validate RNAseq results, and consistent with the transcriptomic data, the expression level of *nlrp3* in YC4W was significantly increased compared with CON (**Figure 4C**). IL-1β was cleaved and secreted after NLRP3 inflammasome activation (46). Thus, the intestinal IL-1β was assessed by ELISA, Western blot, and immunohistochemical analysis. The results suggested that IL-1β significantly increased in the intestine of zebrafish administrated YC for 4 weeks, while YC1W showed no significant difference compared with CON (**Figures 4D–F**). Taken together, 4-week YC administration promoted the activation of NLRP3 inflammasome and increased the secretion of pro-inflammatory cytokine IL-1β.

### The Protective Effect of YC Against *A. hydrophila* Infection Is Mirrored by Sodium Butyrate Addition

**Figure 5:**
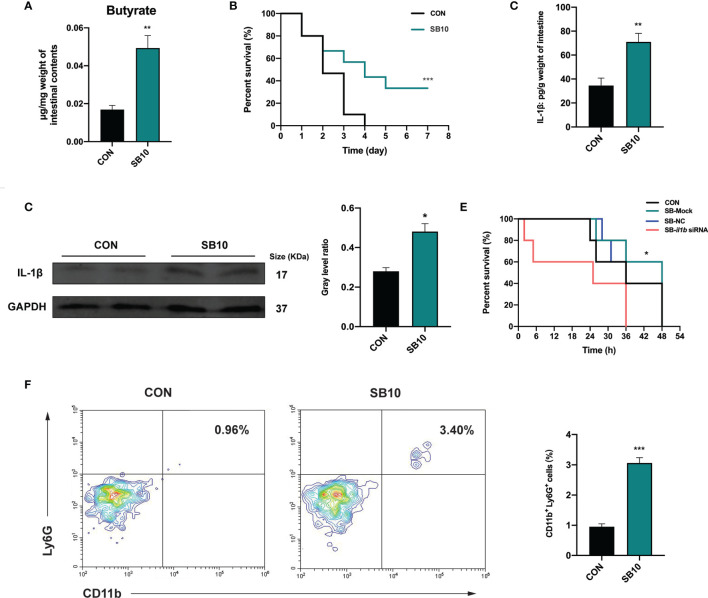
Sodium butyrate addition shows similar protective effect of *P. pentosaceus* YC in pathogen resistance on zebrafish. **(A)** Intestinal butyrate levels of zebrafish fed on commercial diet supplemented with 0 mmol/kg (CON) and 10 mmol/kg (SB10) sodium butyrate (*n* = 3). **(B)** Survival curve of zebrafish from CON and SB10 groups following 10^8^ CFU/ml *A. hydrophila* challenge. Each curve represents the sum of three independent tanks. ***p < 0.001 by Mantel–Cox test. **(C)** ELISA analysis of intestinal IL-1β level of zebrafish from CON and SB10 groups (*n* = 6). **(D)** Representative Western blot of intestinal IL-1β of zebrafish from the CON and SB10 group. **(E)** Survival curve of zebrafish following 10^9^ CFU/ml *A. hydrophila* challenge. Zebrafish were pretreated with 10 mmol/kg sodium butyrate for 1 week and then gavaged with PBS (SB-Mock), 50 µM non-targeting (SB-NC), or *il1b* (SB-*il1b* siRNA) siRNA. Asterisks indicate significant differences between SB-Mock and SB-*il1b* siRNA. *p < 0.05 by Mantel–Cox test. **(F)** Flow cytometry analysis of zebrafish intestinal neutrophils stained with anti-CD11b and anti-Ly6G antibodies (*n* = 3). Numbers indicate the percentage of CD11b^+^Ly6G^+^ neutrophils in intestinal cells. Data are represented as mean ± SEM. *p < 0.05; **p < 0.01; ***p < 0.001 by Student’s *t*-test.

To determine whether the accumulation of butyrate in the intestine was the main factor of YC, zebrafish were fed with a commercial diet supplemented with sodium butyrate (SB) in three different concentrations to imitate the intestinal butyrate level induced by 4-week YC administration. The result indicated that 1 week of 10 mmol/kg SB (SB10) addition enhanced the intestinal butyrate to a comparable level of 4-week YC administration (**Figure 5A** and **Figure S3A**) and then a challenge test with *A. hydrophila* was conducted. Consistent with the result of 4-week YC administration, 10 mmol/kg SB addition significantly improved the survival rate of zebrafish challenged with *A. hydrophila* compared to CON (**Figure 5B**). We measured the intestinal IL-1β in SB10 to verify the activation of NLRP3 inflammasome. Similarly, ELISA and Western blot results showed that 10 mmol/kg SB significantly enhanced the intestinal IL-1β compared to CON (**Figures 5C, D**).

To further determine whether the enhanced IL-1β induced by NLRP3 inflammasome activation contributed to the pathogen resistance effect of YC, an *in vivo* siRNA to knock down *il1b* mRNA was conducted in SB10-treated zebrafish according to the previous research (47). We found that zebrafish gavaged with 50 µM *il1b* siRNA1 (Target site1) for 24 h exhibited lower *il1b* expression level compared to zebrafish gavaged with PBS (Mock) and non-targeting (NC) siRNA (**Figure S4**). Zebrafish gavaged with 50 µM *il1b* siRNA1 for 24 h were used for a 10^9^ CFU/ml *A. hydrophila* challenge. The results showed that the anti-infection effect of SB10 addition was eliminated by *il1b* interference (SB-*il1b* siRNA) (**Figure 5E**). These data indicated that 10 mmol/kg sodium butyrate addition improved the resistance of zebrafish against *A. hydrophila* by enhancing the intestinal level of pro-inflammatory cytokines IL-1β.

### The Enhanced Intestinal Butyrate Induced by 4-Week YC Administration Increases the Recruitment of Neutrophils *In Vivo*

The influence of dietary sodium butyrate supplementation on the recruitment of neutrophils in the intestine was determined. The result showed a significant increase in the percentage of intestinal CD11b^+^ Ly6G^+^ neutrophils in SB10 compared to CON (**Figure 5F**).

**Figure 6:**
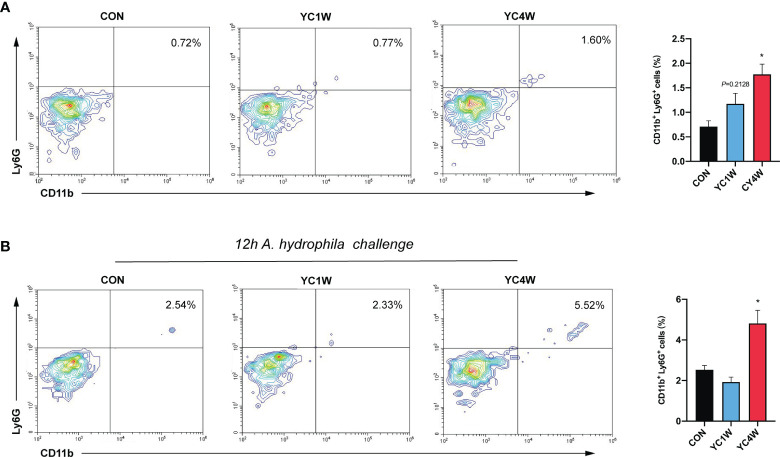
Four-week *P. pentosaceus* YC administration increases the intestinal neutrophils of zebrafish and enhances the recruitment of neutrophils under pathogen infection. **(A)** Flow cytometry analysis of intestinal neutrophils of zebrafish from CON, YC1W, and YC4W groups (*n* = 3). **(B)** Flow cytometry analysis of intestinal neutrophils of zebrafish from CON, YC1W, and YC4W group. All zebrafish were challenged with 10^8^ CFU/ml *A. hydrophila* for 12 h before intestine collection (*n* = 3). Numbers indicate the percentage CD11b^+^Ly6G^+^ neutrophils in intestinal cells. Data are represented as mean ± SEM. *p < 0.05 by one-way analysis of variance followed by Dunnett’s multiple comparisons test.

The migration of intestinal neutrophils was measured in zebrafish treated with YC, and the results indicated that the 4-week YC administration significantly enhanced the percentage of neutrophils in the intestine compared with CON, while YC1W showed no difference with CON (**Figure 6A**). Zebrafish treated with YC were further challenged with 10^8^ CFU/ml *A. hydrophila* for 12 h and the intestinal neutrophil percentage was analyzed. Consistent with the previous finding, the intestinal neutrophil percentage of YC4W was significantly higher than CON and YC1W (**Figure 6B**). All these results suggested that the increased level of butyrate induced by 4-week YC administration raised the percentage of intestinal neutrophils and endued the zebrafish with *A. hydrophila* resistance.

## Discussion

Probiotic administration is a potential strategy against enteric pathogen infection. Different administration period of probiotics influenced their effectiveness, but the underlying mechanism remains unclear. In the present study, 3- or 4-week YC administration increased the pathogen resistance while 1- or 2-week YC administration did not, suggesting that the mechanism by which *P. pentosaceus* competes the pathogen is more complicated than secreting bacteriocin.

Considering the close relationship between the intestinal microbiota and the host immunity, intestinal bacteria in CON, YC1W, and YC4W were detected. We found that except for the bacteria related to SCFA production (ASV2299, 4821, and 1397), the abundance of ASV 4510 (*Hydrogenophaga*) also increased significantly in YC4W (**Figure 2D**). *Hydrogenophaga* is proved to produce poly-3-hydroxybutyrate (PHB) *in vitro* (48). In order to identify whether PHB was involved in the *A. hydrophila* resistance effect, 0.5%, 1%, or 2% of PHB were used to feed zebrafish for 7 days, but no significant difference in survival rate was detected in *A. hydrophila* challenge (**Figure S5**), suggesting that PHB may not be the key effector in the present study.

The results of 16S rRNA gene sequencing and gut SCFA determination showed that butyrate is the differential metabolite between CON and YC4W. Butyrate is an important immune regulator and is reported to suppress inflammation by inhibiting histone deacetylase, NF-κB, and Dact3 (49, 50). Butyrate has also been reported to promote inflammation by increasing the production of IL-1β (51). In the present study, 10 mmol/kg sodium butyrate (SB) raised the survival rate of *A. hydrophila* challenge test and increased the IL-1β production in zebrafish intestine compared with CON, while the 20 and 40 mmol/kg SB treatment group showed no difference with CON (**Figures S3B, C**). A previous study reported that a high concentration of butyrate decreased the pH of the culture medium and inhibited the growth of gut resident bacteria (52). In our study, higher concentrations of butyrate (20 or 40 mmol/kg SB) did not affect IL-1β production, suggesting that the immunomodulation effect of butyrate is multifaceted. Another study also found that a high dose of butyrate (8 mM) would increase the permeability of Caco-2 cell monolayer model while a low dose (2 mM) decreased the permeability (53), indicating that the effect of butyrate is determined by the concentration. IL-1β is a pro-inflammatory factor and research has proved that the secretion of IL-1β may promote the intestinal inflammasome and damage the intestine (54). However, we did not find significant difference between CON and YC4W at the histological level (**Figure S6**).

Furthermore, we found that the administration duration is a key factor for the beneficial effect of probiotics. A study on patients with irritable bowel syndrome (IBS) showed that treatment duration influenced the efficiency of probiotics (55), but the underlying mechanism was not demonstrated. In our study, 3- or 4-week YC treatment had a higher survival rate than 1- or 2-week treatment post *A. hydrophila* challenge test. The possible reason is that the shorter administration duration of YC could not efficiently increase the intestinal butyrate content, which influenced the protective effect of *P. pentosaceus.*

IgM exerts an important role in responding to pathogens both in systemic and mucosal compartments (45). However, there is no difference in intestinal IgM levels between CON and YC4W. PRR activation is important in recognizing and limiting microbes at the beginning of the infection. In the present study, we found that NLRP3 inflammasome is involved in PRR-related pathways and the NOD-like receptor signaling pathway. Our results revealed that 4-week YC administration promoted the production of IL-1β *via* NLRP3 inflammasome activation. Inflammasomes are emerging as critical regulators of the innate immune response and the activation of NLRP3 inflammasome plays an important role in pathogen clearance and gut microbiota homeostasis (56). For example, Nlrp3^−/−^ mice were reported to exhibit decreased antimicrobial capacity (57). Inflammasomes were usually activated by pathogenic bacteria, but a recent study reported that probiotic *E. faecium* promoted the activation of NLRP3 inflammasome (58). *E. faecium* significantly evaluated pro-inflammatory cytokine IL-1β in the jejunum and ileum of 29-day-old piglets which resulted in the higher resistance against enterotoxigenic *E. coli*, but the causal relationship between *E. coli* resistance and NLRP3 activation remained unclear (58). In the present study, we found that the enhanced intestinal butyrate induced by YC administration increased IL-1β production and raised intestinal neutrophil level, which contributed to the improved resistance against *A. hydrophila* infection. These results suggest that promoting host inflammation is one potential way for probiotics to provide their protective effects against pathogens. Although IL-1β plays an important role in pathogen infection, overexpression of IL-1β may also cause severe gastric inflammasome to the host (59). Previous research reported that neutropenic mice that cannot recruit neutrophils efficiently were more susceptible to *A. hydrophila* infection (60), and consistent with their work, we found that the increased neutrophil recruitment in the intestine was related to the enhanced pathogen resistance in zebrafish.

The present study provides new insight into the protective effect of probiotic *P. pentosaceus* by upregulating the host inflammation level, which is a potential strategy against pathogens. The functional mechanism of probiotics determines the administration duration, and the level of microbial metabolites, butyrate, is a determining factor for *P. pentosaceus* exerting their beneficial effect.

## Data Availability Statement

The datasets presented in this study can be found in online repositories. The names of the repository/repositories and accession number(s) can be found at: https://www.ncbi.nlm.nih.gov/bioproject/PRJNA674514; https://www.ncbi.nlm.nih.gov/bioproject/PRJNA674905.

## Ethics Statement

This work was approved by the Committee on the Ethics of Animal Experiments of East China Normal University (assurance no. F20201002).

## Author Contributions

CS and M-LZ designed research. CS conducted most of the experiments. CS and M-LZ analyzed data. ML, ZL, and RX contributed to YC isolation and identification. CS and M-LZ drafted the paper. ML, FQ, Z-YD, and M-LZ revised the paper. All authors contributed to the article and approved the submitted version.

## Funding

This work was supported by the National Key Research and Development Program of China (grant number 2019YFE0115000) and the National Natural Science Foundation of China (grant numbers 31972798 and 31672668).

## Conflict of Interest

The authors declare that the research was conducted in the absence of any commercial or financial relationships that could be construed as a potential conflict of interest.

## Publisher’s Note

All claims expressed in this article are solely those of the authors and do not necessarily represent those of their affiliated organizations, or those of the publisher, the editors and the reviewers. Any product that may be evaluated in this article, or claim that may be made by its manufacturer, is not guaranteed or endorsed by the publisher.
