# Supplementary material for: Pediococcus pentosaceus Enhances Host Resistance Against Pathogen by Increasing IL-1β Production: Understanding Probiotic Effectiveness and Administration Duration
Source: Front Immunol. 2021 Nov 26;12:766401. doi: 10.3389/fimmu.2021.766401 (PMC8662542; doi:10.3389/fimmu.2021.766401)
Supplement: Supplementary file 1 [file DataSheet_1.docx]

***Supplementary materials and methods***

**Supplementary Figures and Tables**

***
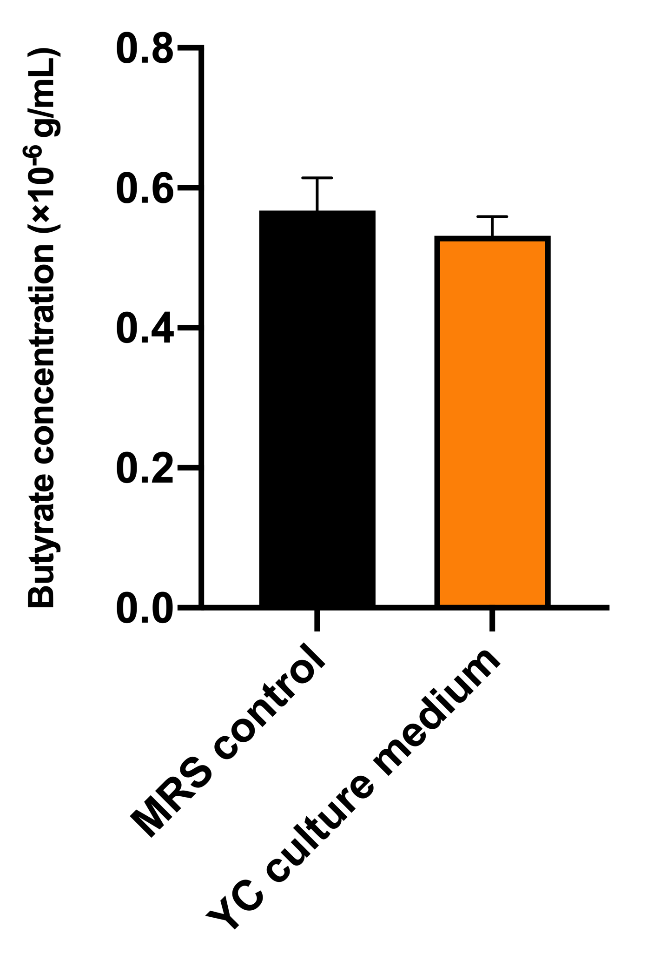
***

**Figure S1.** ***P. pentosaceus* YC did not produce bytyrate in vitro.** The butyrate concentration of YC culture (16 h) medium and MRS control. Data are represented as mean ± SEM.


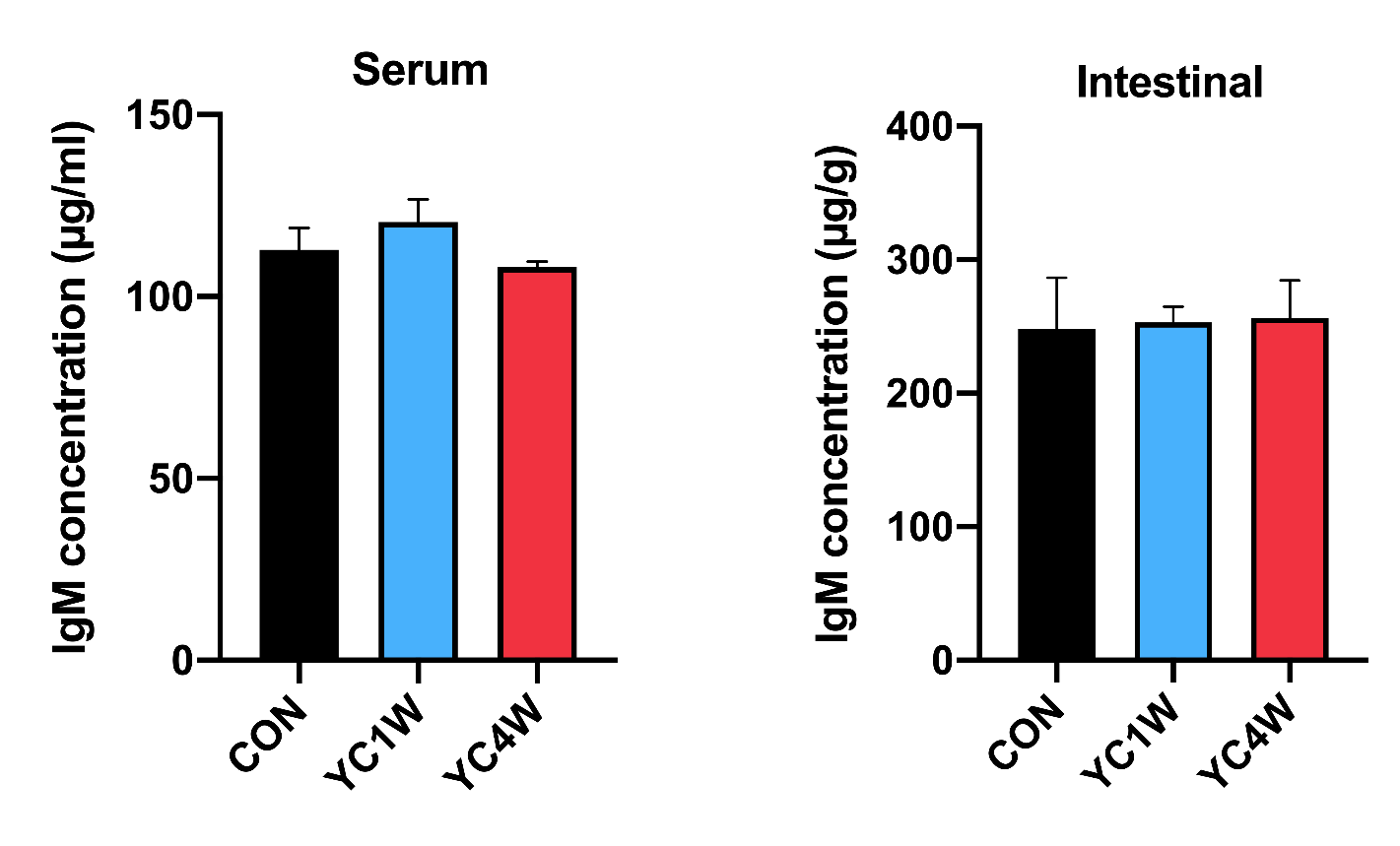


**Figure S2. YC administration has no effect on serum and intestinal IgM of zebrafish.** Serum and intestinal IgM levels of zebrafish from CON, YC1W, and YC4W groups (n=3 or 4). Data are represented as mean ± SEM.


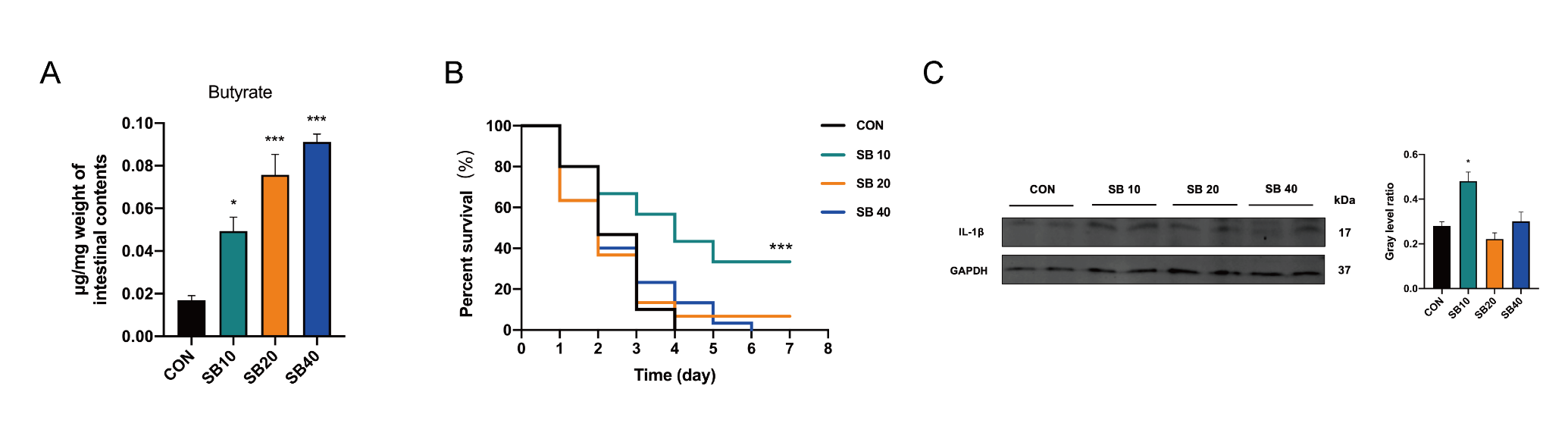


**Figure S3**. **Sodium butyrate addition replicates the effect of *P. pentosaceus* YC on zebrafish.** **(A)** Survival curve of zebrafish following *A. hydrophila* challenge: zebrafish were fed on commercial diet supplemented with 0 mmol/Kg (CON), 10 mmol/Kg (SB10), 20 mmol/Kg (SB20), and 40 mmol/Kg (SB40) sodium butyrate for 1 week. **(B)** Survival curve of zebrafish from CON, SB10, SB20, and SB40 groups following *A. hydrophila* challenge. ****P*<0.001 by Mantel-Cox test. **(C)** Representative western blot of intestinal IL-1β of zebrafish from CON, SB10, SB20, and SB40 groups. Data are represented as mean ± SEM. **P* < 0.05; ***P* < 0.01; ****P* < 0.001 by one-way analysis of variance followed by Dunnett’s multiple comparisons test.


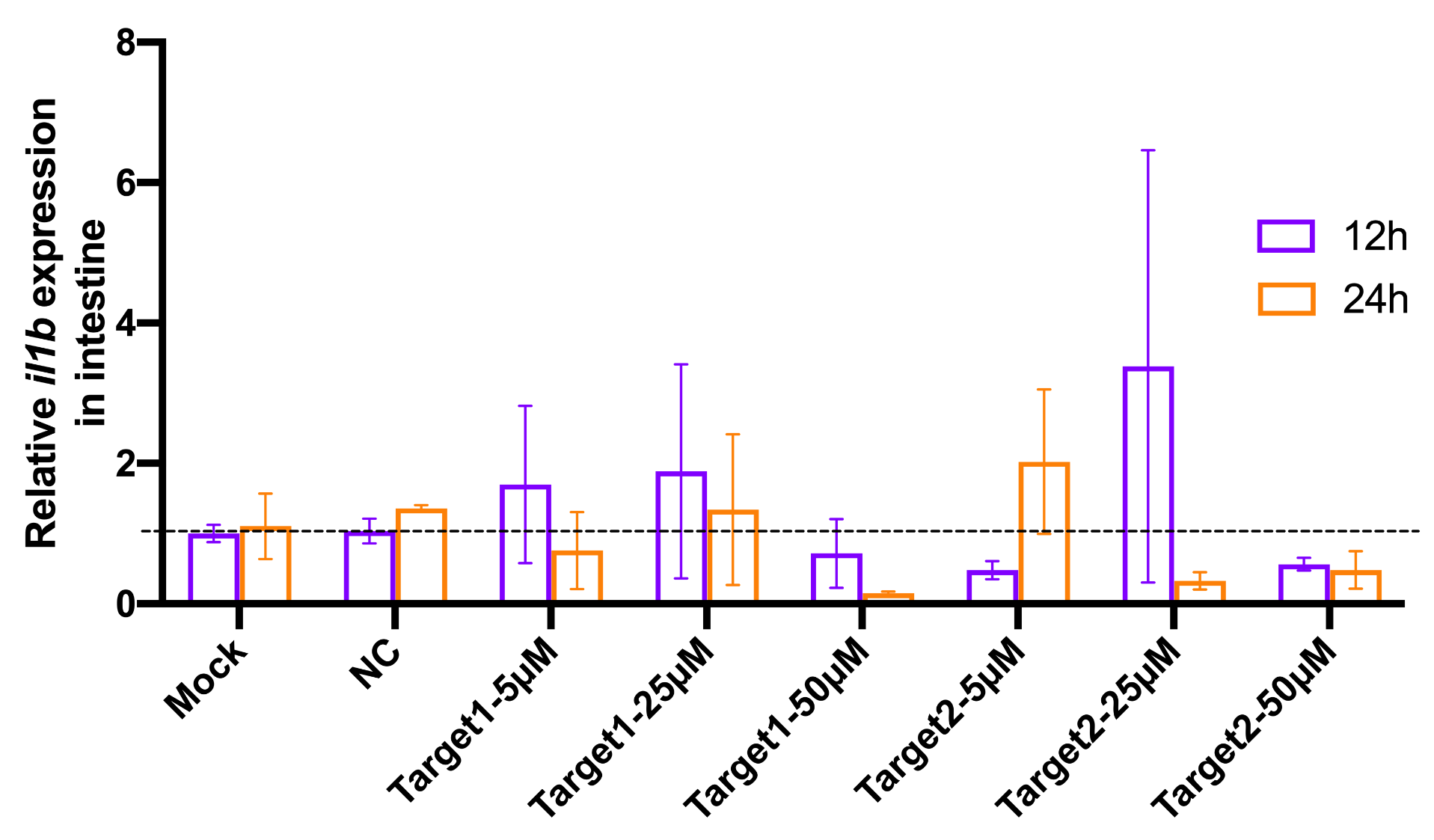


**Figure S4**. ***In vivo* *il1b*-siRNA of zebrafish intestine.** Relative intestinal *il1b* expression level in zebrafish which gavaged with PBS (Mock), non-targeting siRNA (NC), 5 µM, 25 µM, 50 µM *il1b* siRNA1 (Target site1), and 5 µM, 25 µM, 50 µM *il1b* siRNA2 (Target site2) for 12 h or 24 h. Data are represented as mean ± SEM.


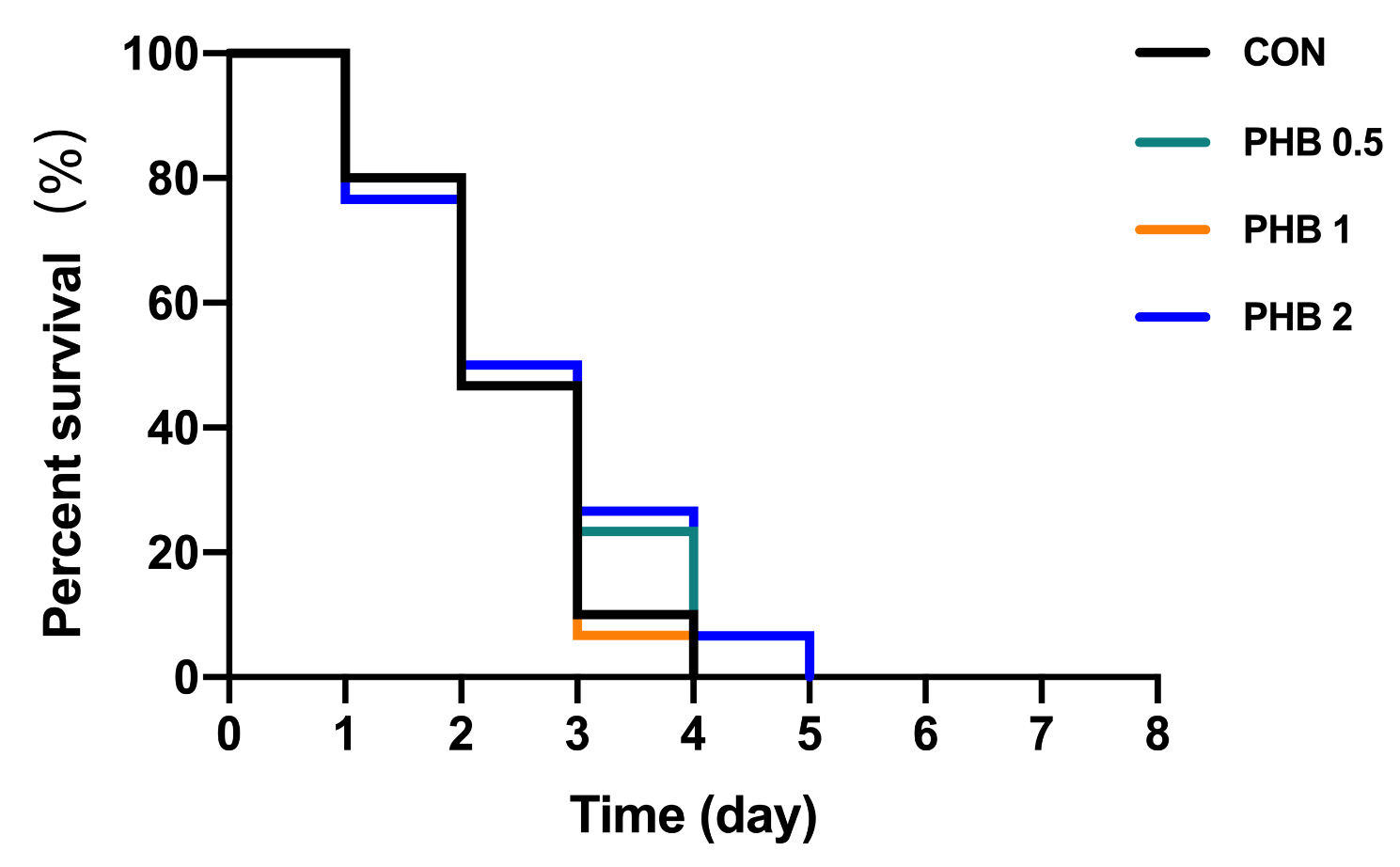


**Figure S5**. **Poly-3-hydroxybutyrate (PHB) addition has no effect on *A. hydrophila* resistance in zebrafish.** Survival curve of zebrafish following 10^8^ CFU/ml *A. hydrophila* challenge: zebrafish were fed on commercial diet supplemented with 0% (CON), 0.5% (PHB 0.5), 1% (PHB 1), and 2% (PHB 2) poly-3-hydroxybutyrate.


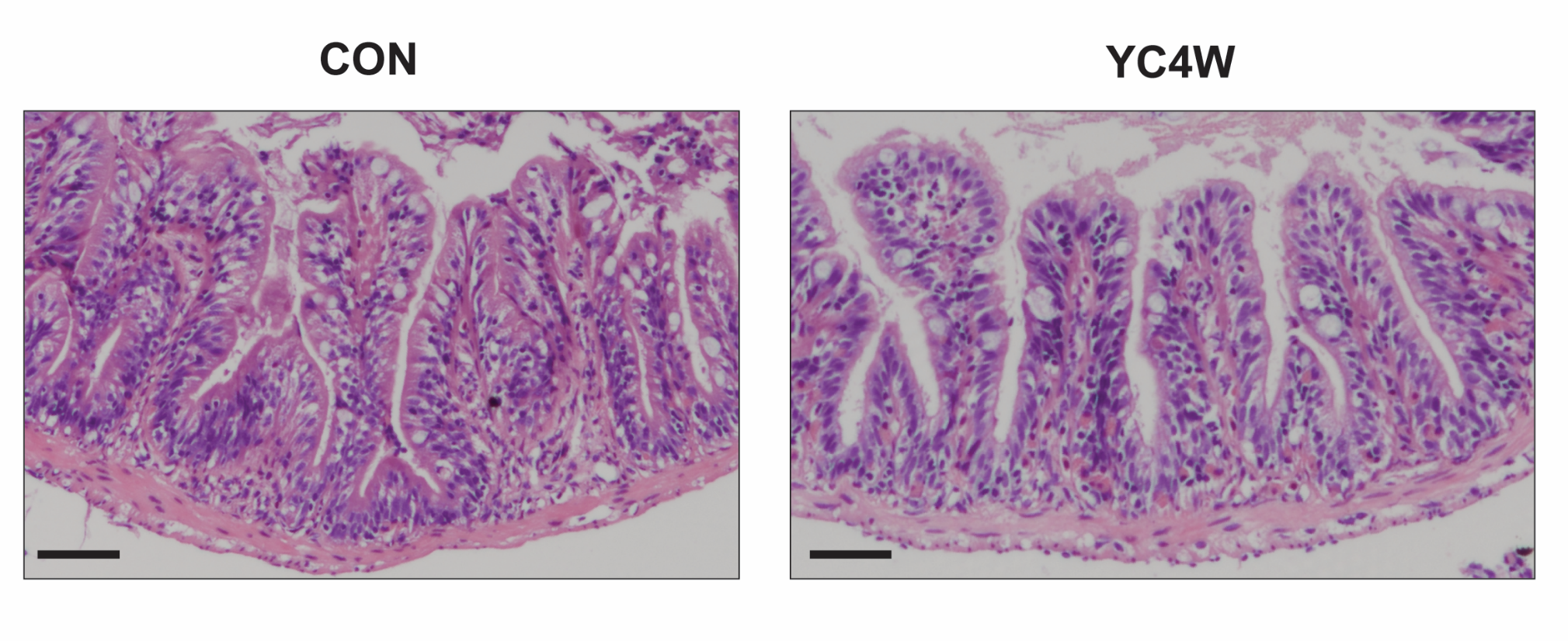


**Figure S6.** **Gut morphology of zebrafish treated with YC for 4 weeks.** Intestinal hematoxylin-eosin(HE) staining of zebrafish that treated with PBS (CON) or YC for 4 weeks, scale bar = 50 μm.

**Table S3** The Primer pairs used for qPCR

|  | **Sequence (5’-3’)** | **Reference** |
| --- | --- | --- |
| ***actb*** | F: CGAGCTGTCTTCCCATCCA  R: TCACCAACGTAGCTGTCTTTCTG | XM_028814403.1 |
| ***ef1a*** | F: CCCCTGGACACAGAGACTTCATC  R: ATACCAGCCTCAAACTCACCGAC | L23807.1 |
| ***gpr109a*** | F: CCTGCACTGAACAGTAAACCG  R: GCATAAATGAAGCTGAGGGCG | XM_017356402.2 |
| ***mct1*** | F: AGGCACGGGATTAGTTGCTG  R: CCAAACCCAGTCCGCCTATG | NM_200085.1 |
| ***nlrp3*** | F: CCTGATGATCTTTTGCCGGG  R: AGAGAGGCTGTGTGAAAGGC | MN088121 |
| ***il1b*** | F: CTGAAATGATGGCATGCGGG  R: TGTAGCTCATTGCAAGCGGA | NM_212844.2 |

**Table S4** *il1b* siRNA information

|  | **Sequence (5’-3’)** | **Chemical modiﬁcation pattern** |
| --- | --- | --- |
| **Target site1** | sense: GAUUCAGUGCCGUCUUACAdTdT  anti-sense: UGUAAGACGGCACUGAAUCdTdT | 2’-O-Me |
| **Target site2** | sense: GCUCCACAUCUCGUACUCAdTdT  anti-sense: UGAGUACGAGAUGUGGAGCdTdT | 2’-O-Me |
| **Non-targeting negative control** | sense: UUCUCCGAACGUGUCACGUdTdT  anti-sense: ACGUGACACGUUCGGAGAAdTdT | 2’-O-Me |

**Table S5** Formulation of the experimental diet

| **Dietary ingredient** | **%** |
| --- | --- |
| **Total fat** | 8 |
| **Total protein** | 52 |
| **Total fiber** | 3 |
| **Ash** | 16.5 |
| **Moisture** | 12 |
| **Calcium** | 5 |
| **Phosphorus** | 1 |
| **Lysine** | 2.5 |

**Supplementary Methods**

## Zebrafish husbandry and experimental diet

For poly-3-hydroxybutyrate (PHB) (Sigma, 363502-10G) supplementation trial, zebrafish were fed with commercial diet (Shengsuo, China) supplemented 0.5% poly-3-hydroxybutyrate (PHB 0.5%), 1% poly-3-hydroxybutyrate (PHB 1%) and 2% poly-3-hydroxybutyrate (PHB 2%) for 7 days. Zebrafish fed on a diet with no additive was designed as control group. During the feeding period of all trial, zebrafish were fed with the diets at a ratio of 4% of their average body weight twice daily.

## ELISA

Serum and intestine of zebrafish from each group were collected, and the level of IgM (QChengBio, QC-IgM-Fi) was determined according to manufacturer’s instructions.

## Gut morphology

Three fish per tank were anesthetized using MS-222 (Sigma, USA) and the anterior intestine segments were fixed in 4% paraformaldehyde for 24 h. Then the paraformaldehyde-fixed samples were embedded in parafﬁn and staining using the methods described before(Zhou et al., 2018).

**References**

Zhou, L., Limbu, S.M., Shen, M., Zhai, W., Qiao, F., He, A., Du, Z.-Y., and Zhang, M. (2018). Environmental concentrations of antibiotics impair zebrafish gut health. Environmental Pollution *235*, 245-254.
